# Oxamate, an LDHA Inhibitor, Inhibits Stemness, Including EMT and High DNA Repair Ability, Induces Senescence, and Exhibits Radiosensitizing Effects in Glioblastoma Cells

**DOI:** 10.3390/ijms26125710

**Published:** 2025-06-14

**Authors:** Takuma Hashimoto, Go Ushikubo, Naoya Arao, Khaled Hatabi, Kazuki Tsubota, Yoshio Hosoi

**Affiliations:** Laboratory of Radiation Biology, Tohoku University School of Medicine, 2-1 Seiryo-machi, Aoba-ku, Sendai 980-8575, Miyagi, Japan; t.hashimoto@tohoku.ac.jp (T.H.); hatabi.khaled.s4@dc.tohoku.ac.jp (K.H.);

**Keywords:** radiation sensitivity, Oxamate, glycolysis, LDH inhibitor, senescence, DNA repair, apoptosis

## Abstract

Enhancement of glycolysis has been reported in tumor cells, and it is believed that this enhancement is important for maintaining the stemness of tumor cells and contributes to malignant phenotypes. Here, we investigated the effects of Oxamate, which inhibits glycolysis by blocking the conversion of pyruvate to lactate, on radiosensitivity and its molecular mechanisms in T98G glioblastoma cells. Oxamate significantly enhanced radiosensitivity by delaying DNA repair, as indicated by the persistence of γ-H2AX foci up to four days post-irradiation. Mechanistically, Oxamate suppressed the expression and phosphorylation of key DNA repair factors. Furthermore, Oxamate induced apoptosis and promoted cellular senescence, as evidenced by the accumulation of SA-β-gal and the upregulation of pS15-p53 and p21. In addition, Oxamate downregulated EGFR expression, reduced the levels of stem cell markers, and modulated epithelial–mesenchymal transition (EMT) markers, suggesting a potential suppression of EMT-related pathways. Together, these results demonstrate that Oxamate enhances radiosensitivity in glioblastoma cells through multiple mechanisms, including the inhibition of DNA repair, induction of apoptosis and senescence, and suppression of cancer stem cell properties and EMT. Our findings provide new insights into the potential use of Oxamate as a radiosensitizer and warrant further investigation of its clinical application in glioblastoma therapy.

## 1. Introduction

Enhancement of glycolysis has been widely observed in tumor cells and is thought to play a crucial role in sustaining stemness and promoting malignant phenotypes. The Warburg effect, characterized by an increased glucose uptake and reliance on aerobic glycolysis for ATP production, is a well-established hallmark of cancer cells [1,2,3]. Lactate dehydrogenase (LDH), particularly the LDHA isoform, facilitates this metabolic shift by converting pyruvate to lactate and regenerating NAD^+^ to maintain glycolytic flux [4,5,6]. LDHA is frequently overexpressed in various tumors and correlates with tumor size, clinical stage, and histological grade [7]. In high-grade gliomas, including glioblastoma (GBM), LDHA expression is significantly elevated compared with low-grade gliomas [8,9], and its overexpression has been linked to resistance to radiotherapy and chemotherapy, particularly temozolomide [10].

Recent studies have demonstrated that Oxamate, a glycolytic inhibitor targeting LDHA, exerts anti-tumor effects in several cancers, including gastric and lung malignancies [11,12,13]. Oxamate suppresses ATP production, induces mitochondrial dysfunction, elevates reactive oxygen species (ROS), and downregulates anti-apoptotic proteins such as Bcl-2 [13,14]. Moreover, Oxamate has been reported to enhance radiosensitivity in GBM cells [15]. However, the molecular basis of this radiosensitizing effect remains unclear. In this study, we investigated how LDHA inhibition by Oxamate affects radiosensitivity in glioblastoma cells, focusing on DNA repair, stemness, and epithelial–mesenchymal transition (EMT)-related markers.

Radiation therapy exerts its cytotoxic effects primarily through the induction of DNA double-strand breaks (DSBs), making efficient DSB repair a critical determinant of radiosensitivity [16]. Defective DSB repair has sensitized cancer cells to radiation [17]. We, therefore, hypothesized that Oxamate may interfere with the DSB repair process in glioblastoma cells following irradiation. DSBs are repaired through two main pathways—homologous recombination (HR) and non-homologous end joining (NHEJ) [18,19]—both of which require rapid expression and autophosphorylation-mediated activation of key DNA repair enzymes.

Among these enzymes, the ataxia–telangiectasia mutated (ATM) kinase plays a pivotal role in DSB repair and is frequently mutated in patients with ataxia–telangiectasia, a disorder marked by radiosensitivity [20,21]. Upon DSB formation, histone H2AX is rapidly phosphorylated to form γ-H2AX, which recruits DNA damage response proteins, including 53BP1 [22,23]. These events facilitate the recruitment and activation of ATM, which undergoes autophosphorylation at serine 1981, transitioning from an inactive dimer to an active monomer [24]. Activated ATM then phosphorylates downstream effectors such as Chk2 to promote DNA repair and initiate cell cycle checkpoints [25]. Similarly, the DNA-dependent protein kinase catalytic subunit (DNA-PKcs) is autophosphorylated at serine 2056 and is essential in NHEJ-mediated DSB repair [26]. The activity and expression levels of both ATM and DNA-PKcs have been reported to correlate with radiation response [27], suggesting that targeting these factors could enhance radiosensitivity in glioblastoma.

In this study, we investigated whether Oxamate affects human glioblastoma cells’ proliferation, survival, and morphology. We also aimed to determine its capacity to modulate radioresistance, particularly through regulating DSB repair pathways, with a specific focus on ATM and DNA-PKcs. Additionally, since EMT and cancer stem cell-like properties are recognized contributors to radioresistance [28,29], we examined the effects of Oxamate on these processes. Our findings demonstrate that Oxamate downregulates key DNA repair regulators, delays DSB repair, and enhances radiosensitivity. Moreover, it suppresses EMT and stemness-related markers, supporting the potential of Oxamate as a radiosensitizer and a promising candidate for further clinical investigation in glioblastoma therapy.

## 2. Results

### 2.1. Effects of Oxamate on Proliferative Ability

**Figure 1:**
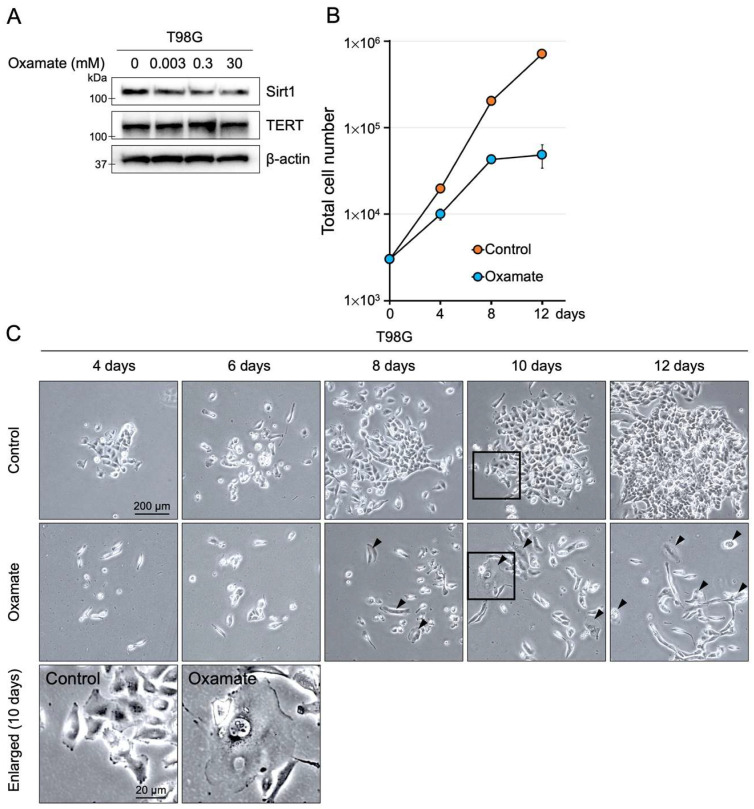
Oxamate reduces the proliferative ability of T98G glioblastoma cells. (**A**) Sirt1 and TERT expression levels in T98G cells treated with Oxamate at concentrations ranging from 0.003 to 30 mM for 24 h. (**B**) Long-term effects of Oxamate on T98G cell proliferation. Cells were seeded at 3000 cells per well in a 6-well plate and cultured overnight. The next day (Day 1), the medium was replaced with 30 mM Oxamate, and cell counts were measured over 12 days. Data are presented as mean ± SD (*n* = 3). (**C**) Representative phase-contrast images showing morphological changes in T98G cells following treatment with 30 mM Oxamate. Black arrowheads indicate giant cells with senescence-like morphology. Enlarged views of the black-boxed areas from the 10-day images are shown below.

We investigated the dose-dependent effects of Oxamate on the proliferative capacity of the human glioblastoma cell line T98G. The highest Oxamate concentration used (30 mM) was selected based on a previous report [15]. T98G cells were treated with Oxamate at concentrations ranging from 0.003 to 30 mM for 24 h. Sirt1 expression, which promotes cell survival and inhibits apoptosis in cancer cells [30], decreased in a dose-dependent manner as the Oxamate concentration increased (Figure 1A). In contrast, Oxamate did not affect telomerase reverse transcriptase (TERT) expression, the catalytic subunit of telomerase. We next assessed the effect of prolonged Oxamate exposure on T98G cell proliferation. When cultured in a medium containing 30 mM Oxamate, T98G cell numbers initially increased over the first 7 to 8 days but then proliferation ceased (Figure 1B). Notably, giant cells with senescence-like morphology appeared after 8 days of treatment (Figure 1C). To determine whether 30 mM Oxamate has similar effects on normal human glial cells, we performed parallel experiments in cultured astrocytes. Oxamate-treated astrocytes exhibited a slight reduction in cell number compared with controls, but this difference was not statistically significant, and cells continued to proliferate (Figure S1A,B). Furthermore, giant cells were not induced in astrocytes. We also performed an MTT assay on day 8 as an indicator of metabolic activity. Oxamate treatment significantly reduced the MTT signal in T98G cells compared with controls, whereas no significant change was observed in astrocytes (Figure S1C). These findings suggest that Oxamate suppresses glioblastoma cell proliferation, coinciding with reduced Sirt1 expression, and may induce a senescence-like state under prolonged exposure while having minimal impact on normal human astrocytes.

### 2.2. Oxamate Induces Cellular Senescence

**Figure 2:**
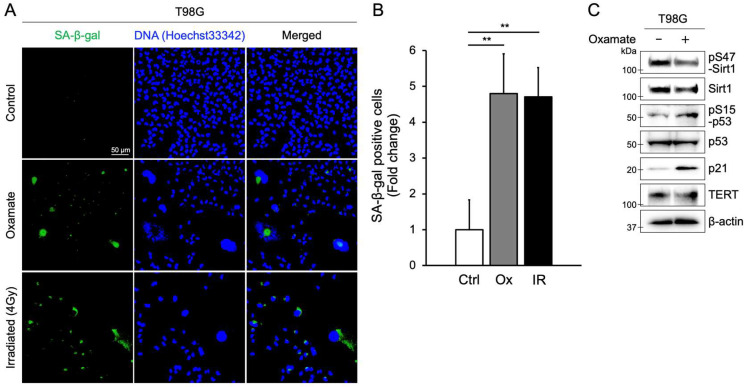
Oxamate induces cellular senescence. (**A**) Representative fluorescence microscopy images of SA-β-gal staining in three treatment groups. Green fluorescence indicates SA-β-gal activity and blue fluorescence represents nuclear staining with Hoechst 33342. (**B**) Fold change in SA-β-gal-positive cells relative to the control group. Values are presented as fold change compared with untreated controls. Values are presented as mean ± SD (*n* = 3). Statistical significance was determined using the Tukey–Kramer test, ** *p* < 0.01 (Ctrl vs. Ox: *p* = 0.015, Ctrl vs. IR: *p* = 0.017). Ctrl—control; Ox—Oxamate; IR—irradiation (4 Gy). (**C**) Expression and/or phosphorylation levels of Sirt1, p53, p21, and TERT in T98G cells after 8 days of treatment with 30 mM Oxamate.

As shown in Figure 1C, prolonged Oxamate exposure led to the emergence of giant cells, a hallmark of senescence-like morphology [31]. To confirm that Oxamate induces cellular senescence, we measured senescence-associated β-galactosidase (SA-β-gal) accumulation, a widely used senescence marker [32]. The level of SA-β-gal-positive cells after 8 days of treatment with 30 mM Oxamate was significantly higher than that of the untreated control group, as indicated by a fold change relative to the control (Figure 2A,B). Radiation exposure can also produce senescence-like giant cells [33]; indeed, the proportion of SA-β-gal-positive cells following Oxamate treatment was similar to that observed 8 days after 4 Gy X-ray irradiation (Figure 2A,B). We further assessed senescence by staining for Ki-67, a proliferation marker, the expression of which is markedly reduced when cells exit the cell cycle. In T98G cells treated with Oxamate, the fraction of Ki-67-positive cells was significantly lower than in untreated controls (Figure S2). By contrast, Oxamate treatment did not alter Ki-67 positivity in normal human astrocytes. Because Oxamate reduced both total Sirt1 expression (Figure 1A and Figure 2C) and Sirt1 phosphorylation at serine 47 (pS47-Sirt1) (Figure 2C), we examined downstream targets negatively regulated by Sirt1—specifically the senescence markers p53 and p21 [34,35]. In Oxamate-treated T98G cells, phosphorylation of p53 at serine 15 (pS15-p53), which is required for p53 activation, increased, and p21 expression was significantly upregulated (Figure 2C). These results demonstrate that Oxamate induces cellular senescence in glioblastoma cells by activating the p53/p21 pathway.

### 2.3. Oxamate Suppresses EGFR Expression and Increases Radiosensitivity

**Figure 3:**
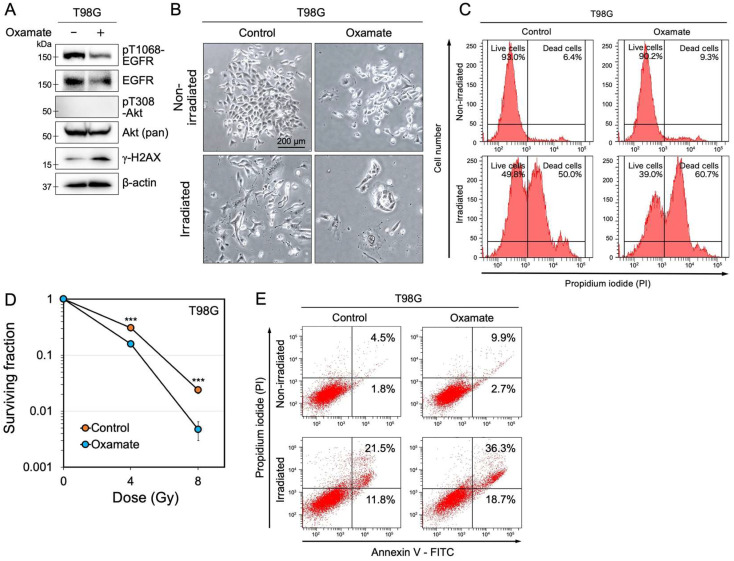
Oxamate suppresses EGFR expression and activity and enhances radiosensitivity. (**A**) Expression and/or phosphorylation levels of EGFR, Akt, and γ-H2AX in T98G cells treated with 30 mM Oxamate for 8 days. (**B**) Representative phase-contrast images of T98G cells after treatment with Oxamate and 8 Gy X-ray irradiation. (**C**) Cell viability assay analyzed by flow cytometry. T98G cells were treated with Oxamate and exposed to 8 Gy X-rays. After 7 days, adherent and floating cells were collected and stained with propidium iodide (PI) for viability analysis. (**D**) Colony formation assay showing the surviving fraction of T98G cells treated with Oxamate and exposed to X-rays (0–8 Gy). After irradiation, cells were cultured for 4 h before plating. Values represent the mean ± SD (*n* = 3). Statistical significance was determined using the Student’s *t*-test (*** *p* < 0.001; 4 Gy: *p* = 0.006, 8 Gy: *p* = 0.003). (**E**) Apoptosis analysis by Annexin V/PI staining. T98G cells were treated with Oxamate, with or without X-ray irradiation (8 Gy), and analyzed for early and late apoptosis using flow cytometry.

Epidermal growth factor receptor (EGFR) is frequently overexpressed in various cancer cells and has been reported to contribute to radioresistance [36,37]. To investigate whether Oxamate affects EGFR expression levels and phosphorylation at key activation sites, we examined total EGFR and phosphorylated EGFR at threonine 1068 (pT1068-EGFR) by Western blotting. We found that total EGFR expression and pT1068-EGFR levels were reduced after Oxamate treatment (Figure 3A). In contrast, the survival signaling molecule Akt showed no significant changes in expression or activation (Figure 3A). In addition, the DNA damage response marker γ-H2AX, which indicates double-strand breaks, was increased after Oxamate treatment (Figure 3A). Given that Oxamate suppressed EGFR expression and activity, we next investigated whether Oxamate enhances radiosensitivity in glioblastoma cells. T98G cells were treated with Oxamate and irradiated with X-rays, and the percentage of dead cells was measured. In the irradiation-only group, the percentage of dead cells was 50.0%, whereas in the combined treatment group (Oxamate + irradiation), the percentage increased to 60.7% (Figure 3B,C). Oxamate treatment alone caused only a slight increase in the percentage of dead cells compared with the control group (6.4% in the control group vs. 9.3% in the Oxamate-treated group). In addition, colony formation assays revealed that the surviving fraction of Oxamate-treated cells was significantly decreased in an X-ray dose-dependent manner compared with control cells (Figure 3D and Figure S2E). These results suggest that Oxamate enhances the radiosensitivity of glioblastoma cells, likely through a reduction in EGFR expression and activity and the accumulation of DNA damage.

### 2.4. Oxamate Induces Apoptosis

EGFR activation has been shown to inhibit apoptosis through the phosphatidylinositol 3-kinase (PI3K)/AKT signaling pathway [38]. In contrast, p53 promotes the expression of many apoptosis-related genes [39]. As shown in Figure 2C and Figure 3A, Oxamate treatment reduced both the expression and activation of EGFR, increasing the expression of p53. Based on these findings, we hypothesized that Oxamate may induce apoptosis in glioblastoma cells. We measured the apoptosis rate in T98G cells to test this hypothesis. In the control group without irradiation, the combined percentage of early and late apoptosis was 6.3% (1.8% early and 4.5% late apoptosis) (Figure 3E). In contrast, Oxamate treatment slightly increased the total apoptosis rate to 12.6% (2.7% early and 9.9% late apoptosis). After irradiation, the control group showed a combined apoptosis rate of 33.3% (11.8% early and 21.5% late apoptosis). However, in the Oxamate + irradiation group, the apoptosis rate increased significantly to 55.5% (18.7% early and 36.8% late apoptosis), representing a 22.2% increase in apoptosis compared with the irradiation-only group. These results suggest that Oxamate enhances radiation-induced apoptosis in glioblastoma cells by suppressing EGFR and activating p53-dependent apoptotic pathways.

### 2.5. Oxamate Suppresses Radiation-Induced Expression and Activation of DNA Repair Factors

**Figure 4:**
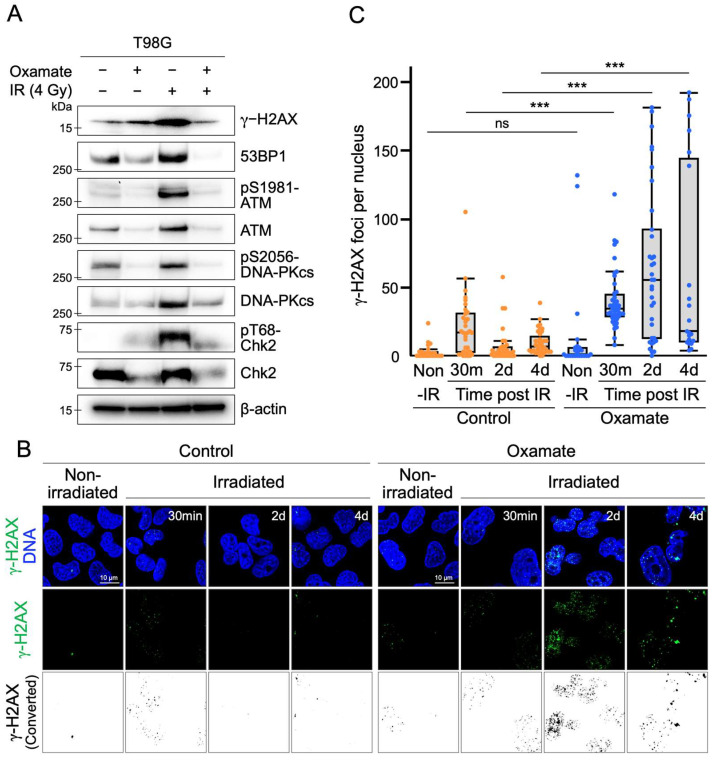
Oxamate suppresses radiation-induced expression and activation of DNA repair factors. (**A**) Western blot analysis showing the expression and/or phosphorylation levels of key DNA repair factors, including γ-H2AX, 53BP1, ATM, Chk2, and DNA-PKcs, in T98G cells treated with Oxamate for 8 days, with or without X-ray irradiation (4 Gy, collected 4 h post-irradiation). (**B**) Representative fluorescence microscopy images illustrating γ-H2AX foci formation under each condition (control or Oxamate, with or without X-ray irradiation). Fluorescence images were processed and converted to binary images to facilitate automated foci counting. (**C**) Quantification of the distribution of γ-H2AX foci per nucleus in each condition. Each dot represents the number of γ-H2AX foci detected in an individual nucleus from T98G cells, under either non-irradiated or irradiated conditions, with or without Oxamate treatment. Orange dots indicate control cells, and blue dots indicate Oxamate-treated cells. Statistical analysis was performed using the Mann–Whitney U test. *** *p* < 0.001; ns—not significant. Detailed *p*-values for group comparisons are as follows: Ctrl non-IR vs. Oxamate non-IR: *p* = 0.48; Ctrl IR 30 min vs. Oxamate IR 30 min: *p* = 2.40 × 10^−5^; Ctrl IR 2 days vs. Oxamate IR 2 days: *p* = 4.94 × 10^−8^; Ctrl IR 4 days vs. Oxamate IR 4 days: *p* = 2.68 × 10^−4^. Sample size: *n* ≥ 21 nuclei per group.

The combination of Oxamate and X-ray irradiation significantly reduced the survival of T98G cells (Figure 3C,D). In addition, this combined treatment induced apoptosis (Figure 3E). Unrepaired or complex DNA damage is a critical factor in inducing apoptosis [40], particularly DNA double-strand breaks (DSBs), which are known to be apoptosis-inducing lesions [41]. Thus, we hypothesized that Oxamate might interfere with the DNA repair response, contributing to the increased radiosensitivity observed in glioblastoma cells. To test this hypothesis, we examined the effect of Oxamate on DNA repair enzymes involved in the DNA damage response. Western blot analysis revealed that Oxamate treatment increased γ-H2AX levels, a marker of DNA double-strand breaks (Figure 3A and Figure 4A). However, key proteins that coordinate with γ-H2AX during the DNA damage response, such as 53BP1 and the downstream DNA repair factors ATM and checkpoint kinase 2 (Chk2), were neither upregulated nor activated following Oxamate treatment (Figure 4A). Similarly, DNA-PKcs, a DNA double-strand break repair enzyme from the same family as ATM, was not induced by Oxamate treatment (Figure 4A). In contrast, radiation exposure alone significantly induced the expression and phosphorylation of these DNA repair proteins. Notably, in the combined treatment group (Oxamate + irradiation), the expression levels and phosphorylation of these proteins were strongly suppressed (Figure 4A). Although γ-H2AX phosphorylation levels in the combined treatment group were comparable to those in the control group, significant reduction in 53BP1 expression was observed, with its band almost completely disappearing compared with the control group (Figure 4A). These results suggest that Oxamate interferes with the activation of the downstream DNA damage response pathway during radiation exposure by reducing γ-H2AX phosphorylation and significantly decreasing 53BP1 expression.

### 2.6. Oxamate Delays DNA Repair

A combination of Oxamate and X-ray irradiation was suggested to negatively regulate the DNA double-strand break (DSB) repair pathway (Figure 4A). To determine whether DNA repair capacity was impaired, we measured the number of γ-H2AX foci as a marker of DNA DSB repair over time. γ-H2AX responds rapidly to radiation exposure [42], and most DNA damage is typically repaired within a few hours to a day [43]. However, it has been reported that γ-H2AX foci do not disappear immediately after complete DNA repair [44]. Therefore, we compared the number of γ-H2AX foci per nucleus between Oxamate-treated and untreated T98G cells at four time points: immediately before irradiation (Non-IR), 30 min after irradiation, 2 days after irradiation, and 4 days after irradiation. In the Non-IR condition, abnormal giant cells with high γ-H2AX foci were observed in the Oxamate-treated group. However, the median number of γ-H2AX foci in the entire population of measured nuclei did not show a significant difference compared with the control group (Figure 4B,C). At 30 min after irradiation, the number of γ-H2AX foci increased in both the control and Oxamate-treated groups. The Oxamate-treated group had a significantly higher number of γ-H2AX foci than the control group. At 2 days post-irradiation, the number of γ-H2AX foci detected in the control group had significantly decreased. In contrast, the Oxamate-treated group showed a heterogeneous population, with some cells exhibiting an abnormally high number of γ-H2AX foci and others exhibiting very few. Notably, the median foci count in the Oxamate-treated group remained significantly higher than in the control group. At 4 days post-irradiation, the median foci count in the Oxamate-treated group also remained significantly higher than in the control group. Giant cells with γ-H2AX were observed in part of the Oxamate-treated group. The population was polarized, with cells showing either abnormally high or very low numbers of γ-H2AX foci. In contrast to the 2-day time point, this polarization was more pronounced. These results suggest that Oxamate induces abnormal γ-H2AX accumulation and delays DNA repair in glioblastoma cells.

### 2.7. Oxamate Suppresses Stemness and EMT in T98G Cells

**Figure 5:**
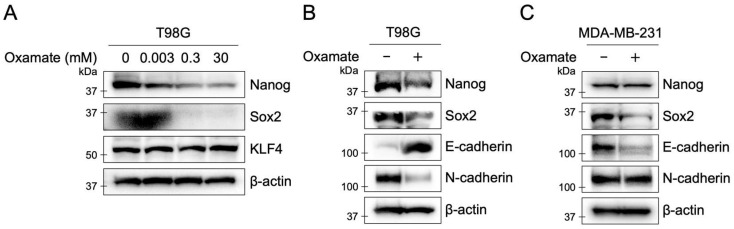
Oxamate suppresses stemness and EMT in T98G cells. (**A**) Expression levels of stem cell markers (Nanog, Sox2, and KLF4) in T98G cells of treatment with different concentrations of Oxamate (0–30 mM). (**B**) Expression levels of stem cell markers and EMT markers (E-cadherin and N-cadherin) in T98G cells after 8 days of treatment with 30 mM Oxamate. (**C**) Expression levels of the same stem cell and EMT markers in MDA-MB-231 cells, a highly malignant breast cancer cell line, after 8 days of treatment with 30 mM Oxamate.

Epithelial–mesenchymal transition (EMT) is known to induce cancer stem cell properties [28], and cancer stem cells exhibit increased resistance to radiotherapy [29]. Therefore, we investigated the effects of Oxamate on EMT and stem cell markers. In T98G cells, Oxamate treatment increased E-cadherin expression and decreased N-cadherin expression, indicating a response opposite to EMT activation (Figure 5). Furthermore, the expression levels of the stem cell markers Nanog and Sox2 decreased dose-dependently with Oxamate treatment. KLF4 expression remained unchanged (Figure 5A,B). In contrast, in the MDA-MB-231 cell line, a highly malignant breast cancer cell line, Oxamate treatment decreased Sox2 and E-cadherin expression, while Nanog and N-cadherin expression remained unchanged (Figure 5C). These results suggest that treatment with Oxamate suppressed EMT and cancer stem cell properties in the human glioblastoma cell.

## 3. Discussion

**Figure 6:**
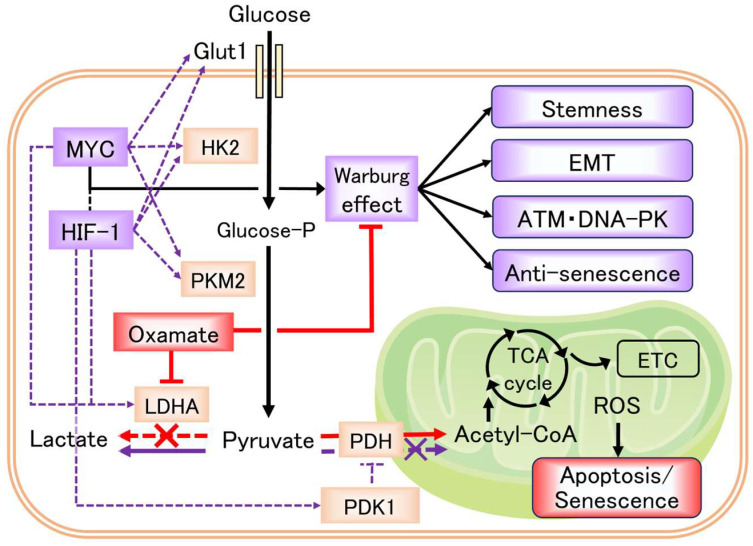
The Warburg effect and the effects of Oxamate in cancer cells. Glucose is metabolized through various reactions by glycolysis, eventually becoming pyruvate. In normal cells under normal oxygen levels, much of this pyruvate enters the mitochondria, where ATP is produced by the tricarboxylic acid (TCA) cycle and the electron transport chain (ETC), providing energy to the cells. However, in cancer cells and cells under hypoxic conditions, MYC and/or HIF-1 control genes involved in glucose metabolism (Glut1, HK2, PKM2, LDHA, PDK1) (purple dotted line), and most of the pyruvate produced by glycolysis does not enter the mitochondria, but instead produces lactate via lactate dehydrogenase (LDHA) (purple solid line). The production of lactate in cancer cells in the presence of oxygen is called the Warburg effect and is related to cancer stemness and tumor growth. Oxamate inhibits LDHA, promoting the use of pyruvate in mitochondria and inhibiting the Warburg effect (red solid line). As a result, Oxamate inhibits stemness, EMT, and ATM/DNA-PK activation and promotes senescence in cancer cells.

In this study, we show that Oxamate, an LDHA inhibitor, enhances radiosensitivity in glioblastoma cells via multiple mechanisms, including delayed DNA repair and increased apoptosis. Additionally, Oxamate suppresses EMT and cancer stem cell markers. We further demonstrate that Oxamate induces cellular senescence in glioblastoma cells and detail the underlying molecular pathways. Table 1 summarizes our key findings, and Figure 6 presents a conceptual model of how Oxamate may alter the Warburg effect and lactate production in cancer cells.

Glycolysis is a critical metabolic pathway that supports the proliferation and survival of cancer cells, and its activation has been associated with treatment resistance in various tumors [4,11,15]. Previous studies reported that Oxamate increases the radiosensitivity of glioma cells [15]. In addition, work using murine melanoma cells and LS174T colorectal adenocarcinoma cells demonstrated that Oxamate reduces radioresistance by inhibiting LDHA activity [45]. Despite these insights, the detailed molecular mechanisms by which Oxamate sensitizes irradiated cancer cells remains unclear.

One of the key findings of our study is that Oxamate impairs the DNA repair process in glioblastoma cells. Under control conditions, radiation induces the expression and phosphorylation of the main proteins involved in the DNA double-strand break (DSB) repair pathway—53BP1, ATM, DNA-PKcs, and Chk2 (Figure 4A). By contrast, in the combined Oxamate plus irradiation group, both expression and phosphorylation of these repair factors were strongly suppressed (Figure 4A), indicating that Oxamate disrupts DSB repair during radiation exposure. Our time-course analysis of γ-H2AX foci supports this conclusion. In the controls, the number of γ-H2AX foci decreased sharply within two days of irradiation, reflecting efficient repair (Figure 4B,C). In Oxamate-treated cells, γ-H2AX foci persisted for at least four days post-irradiation, indicating prolonged DNA damage and delayed repair. Given that unrepaired or complex DNA damage is a potent trigger for apoptosis [40], these results suggest that oxidative repair delays sensitize glioblastoma cells to apoptosis. Indeed, flow cytometry revealed a 22.2% increase in early and late apoptotic cells in the Oxamate plus irradiation group compared with irradiation alone (Figure 3E). This enhanced apoptosis was partly mediated by EGFR suppression and activation of the p53-dependent apoptotic pathway (Figure 2C and Figure 3A). Previous reports have shown that LDHA inhibition can trigger apoptosis via ATP depletion and reactive oxygen species accumulation in cancer cells [13]. Our data further support the model in which Oxamate, by accumulating DNA damage and disrupting survival signaling, drives apoptosis in glioblastoma.

Another notable finding is that Oxamate induces cellular senescence in glioblastoma cells. In T98G, long-term Oxamate treatment produced giant senescence-like cells (Figure 1C) and significantly increased SA-β-gal-positive cells while decreasing Ki-67-positive cells, hallmarks of senescence (Figure 2 and Figure S2A). Radiation itself can also induce senescent giant cells [33], and indeed, SA-β-gal positivity after Oxamate treatment was comparable to that observed eight days after 4 Gy irradiation (Figure 2A,B). At the molecular level, Oxamate reduced both total Sirt1 expression (Figure 1A and Figure 2C) and phosphorylation of Sirt1 at serine 47 (Figure 2C). We then examined downstream targets negatively regulated by Sirt1, specifically the senescence markers p53 and p21 [34,35]. In Oxamate-treated T98G cells, phosphorylation of p53 at serine 15, a key activation site, was enhanced, and p21 expression was upregulated (Figure 2C). These data demonstrate that Oxamate induces senescence via the Sirt1/p53/p21 axis.

We also examined Oxamate’s effects on normal human glial cells, using astrocytes to assess potential toxicity. When cultured with 30 mM Oxamate, astrocytes showed a slight but non-significant reduction in cell number and MTT signals compared with controls, and cells continued to proliferate (Figure S1). Moreover, giant cells did not appear in astrocyte cultures. Likewise, Oxamate did not reduce Ki-67 positivity in astrocytes (Figure S2). Taken together, these findings suggest that Oxamate selectively suppresses glioblastoma proliferation—accompanied by reduced Sirt1 expression and senescence induction—while having minimal impact on normal astrocytes. This differential response likely reflects the metabolic differences between glioblastoma cells and normal glial cells. In glioblastoma, the Warburg effect drives high glycolytic flux, making cells reliant on LDHA for NAD^+^ regeneration and lactate production. Normal glial cells, however, do not exhibit the same degree of glycolytic dependency under normoxic conditions. As a result, LDHA inhibition by Oxamate disproportionately disrupts energy production in cancer cells, supporting the concept that targeting the glycolytic phenotype can selectively impair tumor cells while sparing normal tissues.

On the other hand, our detailed investigation also confirmed that long-term exposure to 30 mM Oxamate alone caused only mild reductions in glioblastoma cell proliferation (Figure 1B and Figure 3C,E). Cell counts plateaued after seven to eight days, likely due to cell division arrest, as evidenced by giant cell formation. Previous studies have shown that other glycolysis inhibitors, such as 2-deoxyglucose, dichloroacetate, and 3-bromopyruvate, exhibit low in vitro toxicity, and Oxamate likewise did not significantly increase cell death or apoptosis in the absence of radiation [46]. Our data reinforce that Oxamate has low basal cytotoxicity yet effectively induces senescence under prolonged exposure through Sirt1/p53/p21 activation (Figure 2C), and its efficacy is further enhanced when combined with radiation.

Beyond DNA repair and senescence, Oxamate also suppressed EMT and cancer stem cell properties. EMT, defined by the loss of epithelial markers and the gain of mesenchymal traits, promotes metastasis, progression, and therapy resistance [47]. Cancer stem cells, which share EMT features, are notoriously radioresistant [28,29]. In T98G cells, Oxamate increased E-cadherin and decreased N-cadherin expression, indicating EMT reversal (Figure 5A,B). Oxamate also dose-dependently reduced the stem cell markers Nanog and Sox2 (Figure 5A,B). These results suggest that Oxamate may enhance radiosensitivity by simultaneously impairing EMT and stemness, thereby reducing glioblastoma’s capacity to resist radiation. Interestingly, Oxamate’s effects on EMT and stem cell markers varied by cell type. In MDA-MB-231 breast cancer cells, Oxamate decreased Sox2 and E-cadherin but did not affect Nanog or N-cadherin (Figure 5C). This variability implies that Oxamate’s impact on metabolic and signaling pathways may depend on tumor context, warranting further studies across diverse cancer models to evaluate its broad-spectrum potential.

A potential limitation of the present study is the absence of in vivo validation of Oxamate’s radiosensitizing effects. Although our in vitro data clearly show delayed DNA repair, enhanced apoptosis and senescence, and suppression of cancer stem cell/EMT markers, these findings must be confirmed in an appropriate animal model before clinical translation. Future work will, therefore, include xenograft experiments in mice to evaluate tumor growth delay, survival benefit, and systemic toxicity when Oxamate is combined with radiation. In parallel, we will conduct pharmacokinetic and tissue-distribution studies to establish dosing regimens that are both effective and safe. Likewise, we recognize that 30 mM Oxamate is a relatively high concentration in vitro. Precedents such as misonidazole and etanidazole demonstrate that strong in vitro radiosensitizing activity does not necessarily translate in vivo—those compounds proved neurotoxic at the doses required for efficacy [48,49]. By contrast, a report suggests that Oxamate may protect normal neurons under ischemic conditions [50]. Nevertheless, before any clinical application can be considered, Oxamate’s potential neurotoxicity and off-target effects must be rigorously evaluated in vivo, alongside detailed pharmacokinetic profiling.

In summary, our findings reveal that Oxamate impairs DNA repair, activates apoptotic and senescence pathways, and inhibits EMT and stemness features in glioblastoma cells, collectively enhancing radiosensitivity. Importantly, Oxamate has minimal effects on normal human astrocytes, suggesting a favorable therapeutic window. These results underscore the promise of targeting glycolysis and, specifically, LDHA to improve radiotherapy outcomes in glioblastoma. Future research should focus on in vivo validation and the potential of Oxamate to overcome radioresistance when used alongside other therapeutic agents.

## 4. Materials and Methods

### 4.1. Cell Culture

The human glioblastoma cell line T98G and the human breast cancer cell line MDA-MB-231 (American Type Culture Collection, VA, USA) were cultured in Dulbecco’s Modified Eagle’s Medium (DMEM, 08456-36, Nacalai Tesque, Kyoto, Japan) supplemented with 10% fetal bovine serum (SH30396.03, Cytiva, Tokyo, Japan) and 1% penicillin–streptomycin mixed solution (09367-34, Nacalai Tesque, Kyoto, Japan). Normal human astrocytes were obtained from ScienCell Research Laboratories, Inc. (Carlsbad, CA, USA) and cultured as recommended by the supplier. Cells were maintained at 37 °C in a humidified atmosphere containing 5% CO_2_. Cell morphology was observed using a phase-contrast microscope (Eclipse Ti2, Nikon, Tokyo, Japan), and images were captured with a Digital Sight 1000 camera (Nikon, Tokyo, Japan).

### 4.2. Chemical

The inhibitor used in this study was Oxamate (Sodium Oxamate, FUJIFILM Wako Pure Chemical Corporation, Osaka, Japan). Oxamate was dissolved in growth medium immediately before use. Twenty-four hours after cell seeding, the medium was replaced with the medium containing the inhibitor.

### 4.3. Proliferation Assay

To assess cell proliferation, cells were seeded at a density of 3000–5000 cells per well in a 6-well plate and cultured overnight. Oxamate treatment was initiated the following day, marking the start of the experiment (Day 1). The assay was conducted for up to 12 days. Cells were washed twice with phosphate-buffered saline (PBS (−)) and detached from the culture surface by trypsinization. Total cell numbers were determined using a particle counter (Coulter Counter Z1, Beckman Coulter, Brea, CA, USA), and dead cells were excluded using the trypan blue dye exclusion method. Cell proliferation was also assessed using an MTT assay kit (23506-80, Nacalai, Kyoto, Japan) following the manufacturer’s instructions. T98G cells or human astrocytes were seeded in 96-well plates at 1 × 10^4^ cells per well in 100 µL of growth medium and incubated for 24 h. Ten microliters of MTT reagent was then added to each well, and plates were incubated for 2 h at 37 °C in 5% CO_2_. After incubation, 5 µL of solubilization solution was added to each well, and absorbance was measured at 570 nm using a microplate reader (Molecular Devices FlexStation3, Molecular Devices, LLC. Tokyo, Japan).

### 4.4. X-Ray Irradiation

X-ray irradiation was performed using an X-ray generator (M-150WE-H, SOFTEX, Kanagawa, Japan). Cells were exposed to doses ranging from 0 to 8 Gy at 130 kV and 8 mA, with a dose rate of 0.564 Gy/min, using a 0.5 mm thick aluminum filter. After irradiation, the cells were returned to an incubator at 37 °C.

### 4.5. Colony Formation Assay

Four hours after irradiation, cells were trypsinized, detached, and counted using a particle counter (Coulter Counter Z1, Beckman Coulter, Brea, CA, USA). The cell suspension was mixed with trypan blue to exclude dead cells. The cell density was adjusted to 150–2500 cells per dish, and the cells were seeded in 60 mm dishes. After incubation at 37 °C for 14–21 days, colonies were fixed with 10% formaldehyde and stained with 0.01% crystal violet. Plating efficiency (PE) was calculated by dividing the number of colonies containing more than 50 cells by the number of cells initially plated. The survival fraction (SF) was determined by dividing the PE of irradiated cells by the PE of non-irradiated control cells.

### 4.6. Western Blotting

Western blotting was performed as previously described [51,52,53]. Protein concentration was quantified using the BCA Protein Assay Kit (Pierce BCA Protein Assay Kit, Thermo Fisher Scientific K.K., Tokyo, Japan) following the manufacturer’s instructions. Samples were prepared with SDS sample buffer containing 1% bromophenol blue and 1% loading dyes and resolved by SDS-PAGE (5–20% gradient gel, E-D520L, ATTO Technology, Tokyo, Japan). Proteins were transferred to a polyvinylidene fluoride (PVDF) membrane (EMD Millipore, Billerica, MA, USA). Membranes were blocked for 60 min with 5% skim milk or PVDF Blocking Reagent (NOF Corporation, Tokyo, Japan), the latter used for phospho-specific antibodies. Membranes were incubated with primary antibodies (1:1000) (Supplementary Table S1) overnight at 4 °C and with secondary antibodies (1:3000) (Supplementary Table S1) for 60 min at room temperature. Antibody diluents were prepared using Can Get Signal Solution 1 & 2 (Toyobo, Osaka, Japan). Detection was performed using chemiluminescent reagents (Chemi-Lumi One Ultra, 11644-40, Nacalai Tesque) and visualized with the ChemiDoc XRS Plus system (Bio-Rad, Hercules, CA, USA). Molecular weights were estimated using Precision Plus Protein Dual Color Standards (Cat: #1610374, batch 64601809; Bio-Rad, Hercules, CA, USA). Original Western blot images are presented in Figure S3.

### 4.7. Flow Cytometry

T98G cells were treated with 30 mM Oxamate for 8 days, followed by X-ray irradiation at a dose of 8 Gy, and then cultured for another 7 days. Both adherent cells and floating cells in the culture supernatant were collected and stained with propidium iodide (PI) (Dojindo Molecular Technologies, Kumamoto, Japan) together with 10 μg/mL RNase A (Nippon Gene, Tokyo, Japan). Cells were incubated at room temperature for 15 min. The Annexin V-FITC Apoptosis Detection Kit (15342–54, Nacalai Tesque, Kyoto, Japan) was used for apoptosis assay per the manufacturer’s protocol. The PI and FITC signals in each cell were analyzed using a FACSCanto II flow cytometer system (Becton, Dickinson and Company, Franklin Lakes, NJ, USA). Gating was performed using SSC-A and FSC-A to exclude doublets and debris from the analysis.

### 4.8. Detection of γH2AX Foci

Detection of γH2AX foci was performed as previously described [54]. Cultured cells on microscope slides (SCS-N04, Matsunami, Japan) were irradiated with 1 Gy using an X-ray generator (SOFTEX). Cells were fixed with 4% formaldehyde for 20 min and permeabilized with 0.5% Triton X-100 for 15 min at various time points: unirradiated (control), 30 min post-irradiation, 2 days post-irradiation, and 4 days post-irradiation. Cells were blocked with 10% goat serum for 1 h and incubated with anti-phospho-histone H2AX (Ser139) antibody clone JBW301 (Merck Millipore, Burlington, MA, USA, 05-636, diluted 1:500) for 1 h, followed by Alexa Fluor 488 anti-mouse IgG (Molecular Probes, A-11001, diluted 1:500) for 1 h. Nuclear DNA was counterstained with DAPI (Invitrogen, Carlsbad, CA, USA). All reactions were conducted at room temperature. Fluorescent signals were visualized using confocal microscopy (LSM800, Carl Zeiss, Oberkochen, Germany). Acquired images were analyzed as AiryScan images using ZEN software (version 2.3 Blue) and binarized using ImageJ software (version 1.54). Noise signals in regions outside the nuclei and incomplete nuclear signals at image edges were excluded from the analysis. Thresholds for image conversion were manually adjusted to achieve the best visual fit between the original and binarized images. The number of γH2AX foci was counted using ImageJ software [55].

### 4.9. Detection of Ki-67 Signals

Cells were cultured on Microscope Glass Slide (SCS-NO4, Matsunami, Osaka, Japan). After being cultured, the cells were fixed with 4% formaldehyde for 20 min and permeabilized with 0.5% Triton X-100 for 15 min. The cells were then blocked with 10% goat serum for 10 min and incubated with anti-Ki-67 primary antibody (#9129, Cell Signaling Technology, Essex County, MA, USA; 1/500 dilution) for 1 h, followed by incubation with Alexa Fluor 488 goat anti-rabbit IgG (Molecular Probes, Eugene, OR, USA. A-11008, 1/500 dilution) for 1 h. Nuclear DNA was counterstained with 4′,6-diamidino-2-phenylindole (DAPI, Dojindo, Kumamoto, Japan). All incubations were performed at room temperature. Fluorescence images were captured on a Zeiss LSM 800 confocal microscope and processed as AiryScan images using ZEN 2.3 (Blue Edition, Carl Zeiss, Oberkochen, Germany) software.

### 4.10. Statistical Analysis

The effects of Oxamate in the SA-β-gal staining assay, MTT assay, γ-H2AX assay, Ki-67 assay, and colony formation assay were evaluated using statistical tests. A two-tailed Student’s *t*-test or a Mann–Whitney U test was used for pairwise comparisons, depending on variance equality, as determined by an F-test. For comparisons among three groups, the Tukey–Kramer test was used to evaluate differences between all possible pairs.

## Figures and Tables

**Table 1:** Summary of Major Findings.

Key Finding	Experimental Assay	Figure Reference
Induction of senescence and apoptosis	Proliferation assay, MTT assay, SA-β-gal staining, Ki-67 staining, Annexin V/PI staining, Western blot (Sirt1, p53, p21)	Figure 1, Figure 2 and Figure 3E, Figures S1 and S2
Delayed DNA repair and enhanced radiosensitivity	Colony formation assay, γ-H2AX foci counting, Western blot (53BP1, ATM, DNA-PKcs)	Figure 3 and Figure 4
Downregulation of stemness markers	Western blot (Nanog, Sox2)	Figure 5A,B
Modulation of EMT proteins	Western blot (E-cadherin, N-cadherin)	Figure 5A,B

## Data Availability

The authors confirm that the data supporting the findings of this study are available within the article.

## Supplementary Materials

The following supporting information can be downloaded at: https://www.mdpi.com/article/10.3390/ijms26125710/s1.
