# Supplementary material for: Oxamate, an LDHA Inhibitor, Inhibits Stemness, Including EMT and High DNA Repair Ability, Induces Senescence, and Exhibits Radiosensitizing Effects in Glioblastoma Cells
Source: Int J Mol Sci. 2025 Jun 14;26(12):5710. doi: 10.3390/ijms26125710 (PMC12193169; doi:10.3390/ijms26125710)
Supplement: Supplementary file 1 [file ijms-26-05710-s001.zip › ijms-3617662-supplementary.pdf]

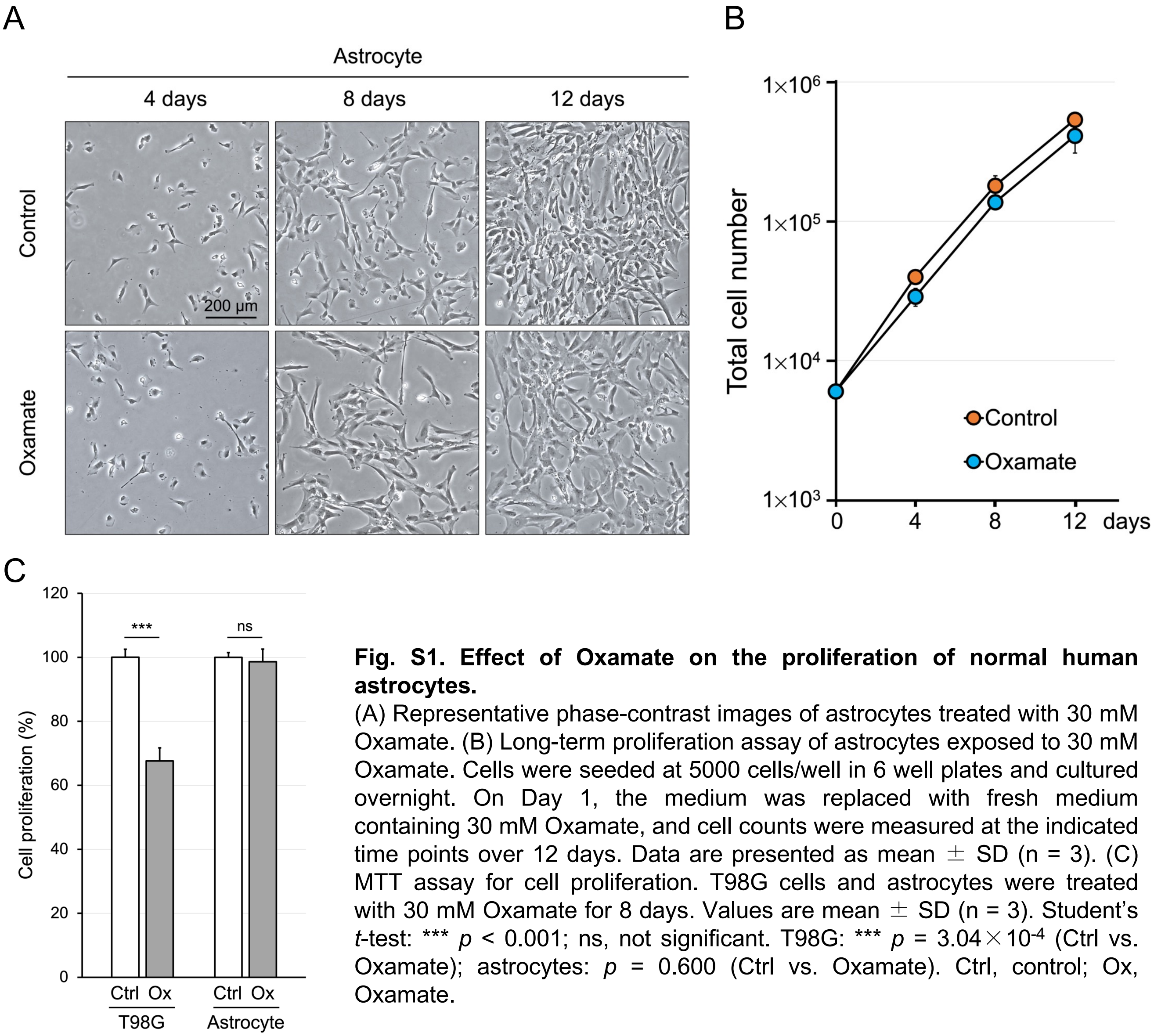

Fig. S1. Effect of Oxamate on the proliferation of normal human astrocytes.
(A) Representative phase-contrast images of astrocytes treated with 30 mM Oxamate. (B) Long-term proliferation assay of astrocytes exposed to 30 mM Oxamate. Cells were seeded at 5000 cells/well in 6 well plates and cultured overnight. On Day 1, the medium was replaced with fresh medium containing 30 mM Oxamate, and cell counts were measured at the indicated time points over 12 days. Data are presented as mean $\pm$ SD (n = 3). (C) MTT assay for cell proliferation. T98G cells and astrocytes were treated with 30 mM Oxamate for 8 days. Values are mean $\pm$ SD (n = 3). Student's *t*-test: *** $p < 0.001$; ns, not significant. T98G: *** $p = 3.04 \times 10^{-4}$ (Ctrl vs. Oxamate); astrocytes: $p = 0.600$ (Ctrl vs. Oxamate). Ctrl, control; Ox, Oxamate.

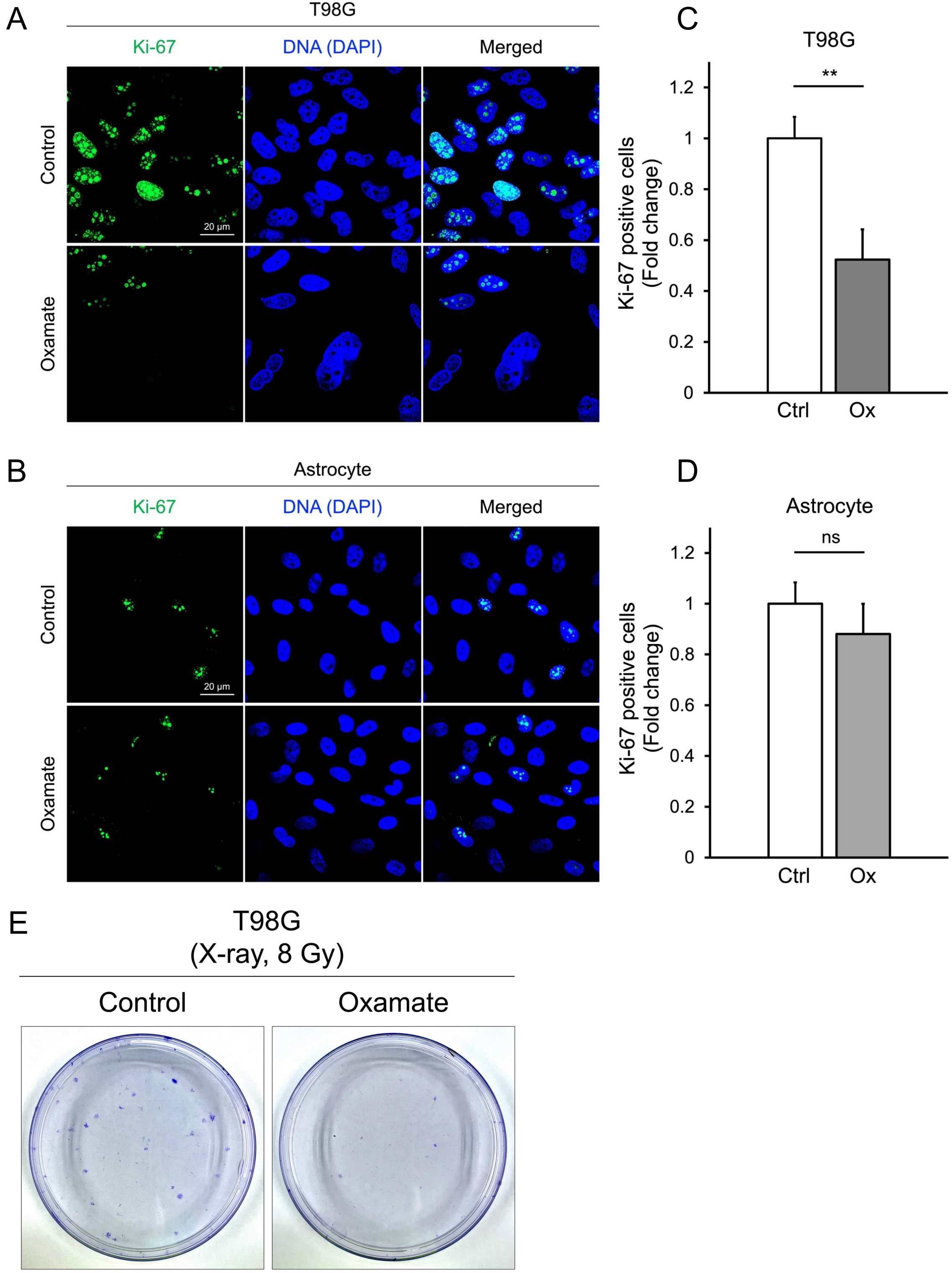

Fig. S2. Effects of Oxamate on Ki-67 and colony formation of T98G cells after irradiation. (A, B) Ki-67 immunofluorescence in T98G cells and astrocytes. Representative confocal images of Ki-67 staining in (A) T98G cells and (B) astrocytes treated with 30 mM Oxamate for 8 days. Green fluorescence indicates Ki-67 and blue fluorescence represents DAPI-stained nuclei. (C, D) Quantification of Ki-67 positive cells. The percentage of Ki-67 positive cells in (C) T98G and (D) astrocyte cultures was calculated from at least three independent fields (total n ≥ 67 nuclei). Values are presented as mean ± SD. Student's *t*-test: ** $p < 0.01$; ns, not significant. T98G (Panel C): $p = 9.80 \times 10^{-3}$ (Ctrl vs. Oxamate); astrocytes (Panel D): $p = 0.434$ (Ctrl vs. Oxamate). (E) Colony formation of T98G cells after Oxamate treatment and irradiation. T98G cells were treated with 30 mM Oxamate for 8 days, then exposed to 8 Gy X-ray. After removal of dead cells, equal numbers of viable cells from the control and Oxamate-treated groups were replated and cultured. Representative phase-contrast images of resulting colonies are shown. Ctrl, control; Ox, Oxamate.

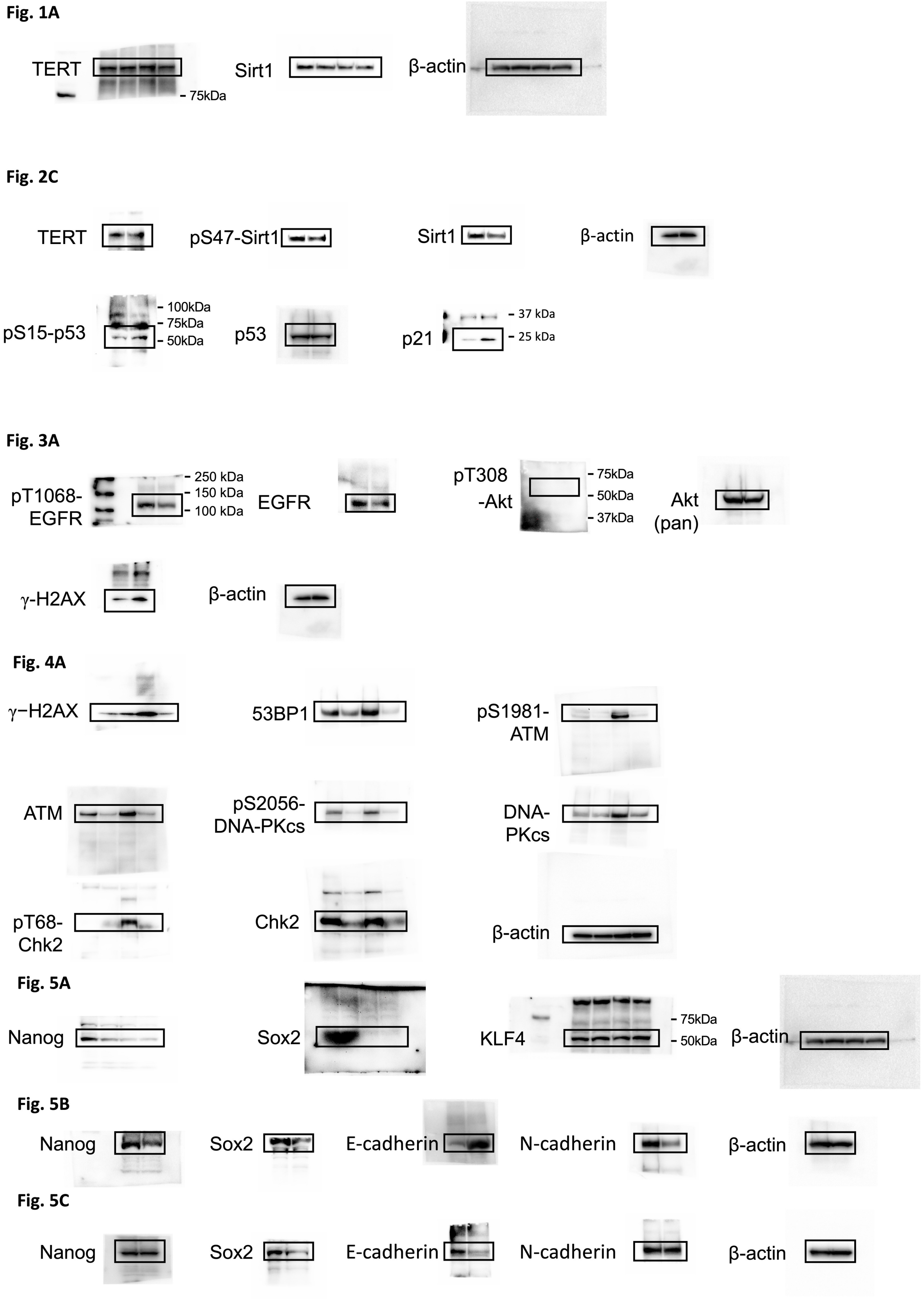

Fig. S3. Original Western blotting data.

Supplementary table S1

List of antibodies for Western blotting

Antibodies	Cat. No.	Vendors
53BP1 antibody	4937S	Cell Signaling Technology
Akt (pan) antibody	4691S	Cell Signaling Technology
ATM antibody	NB100-104	Novus Biologicals
Chk2 antibody	2662S	Cell Signaling Technology
DNA-PKcs antibody	SC-9051	Santa Cruz
E-cadherin antibody	ab40772	Abcam
EGFR antibody	2232S	Cell Signaling Technology
HRP Anti-beta Actin antibody	ab49900	Abcam
KLF4 antibody	ab106629	Abcam
N-cadherin antibody	ab18203	Abcam
Nanog antibody	ab109250	Abcam
p21 antibody	2947S	Cell Signaling Technology
p53 antibody	9282S	Cell Signaling Technology
Phospho-Akt (T308) antibody	9275S	Cell Signaling Technology
Phospho-ATM (S1981) antibody	5883S	Cell Signaling Technology
Phospho-Chk2 (pT68) antibody	2661S	Cell Signaling Technology
Phospho-DNA-PKcs (S2056) antibody	ab124918	Abcam
Phospho-EGFR (Y845) antibody	2231S	Cell Signaling Technology
Phospho-p53 (S15) antibody	9286S	Cell Signaling Technology
Phospho-Sirt1 (S47) antibody	2314S	Cell Signaling Technology
Polyclonal Goat Anti-Mouse immunoglobulins/HRP	P0447	Dako
Polyclonal Swine Anti-Rabbit immunoglobulins/HRP	P0399	Dako
Sirt1 antibody	9475S	Cell Signaling Technology
Sox2 antibody	ab97959	Abcam
TERT antibody	NB110-89471	Novus Biologicals
γ-H2AX antibody	05-636	Sigma-Aldrich
